# Supplementary material for: Climate’s firm grip on glacier ablation in the Cordillera Darwin Icefield, Tierra del Fuego
Source: Nat Commun. 2025 Mar 18;16:2677. doi: 10.1038/s41467-025-57698-6 (PMC11920049; doi:10.1038/s41467-025-57698-6)
Supplement: Supplementary file 1 — Supplementary Information [file 41467_2025_57698_MOESM1_ESM.pdf]

Supplementary material to Temme et al., ‘Climate’s firm grip on glacier ablation in the Cordillera Darwin Icefield, Tierra del Fuego’

Supplementary Figures

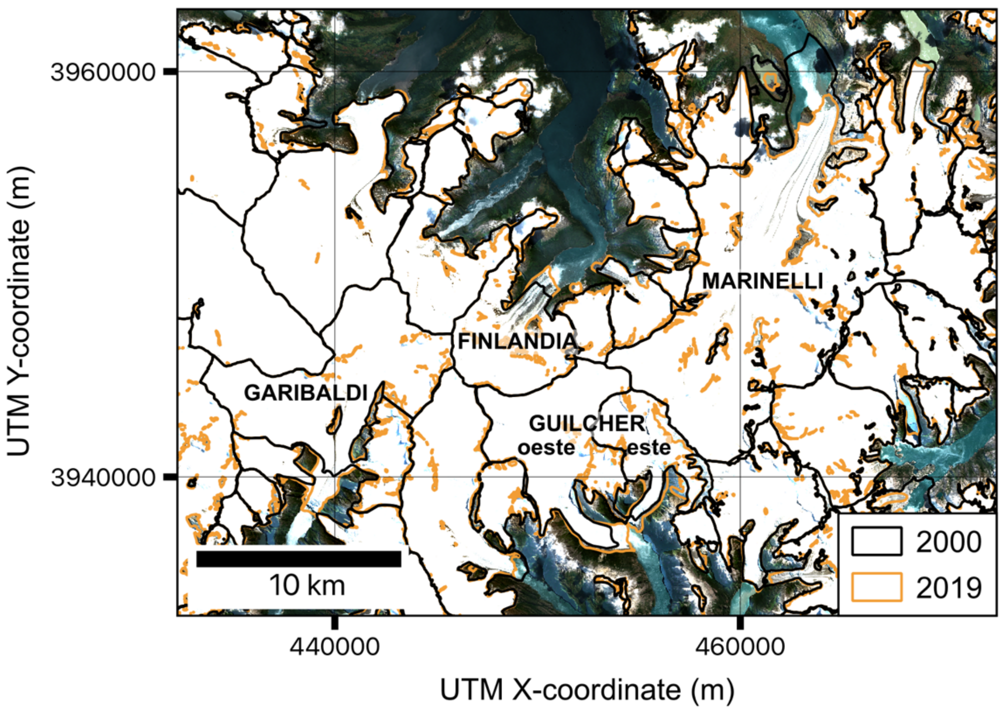

Figure S1: Retreating and advancing glaciers. *Glacier outlines from the Chilean Glacier Inventory for 2000 (Barcaza et al., 2017) and 2019 (DGA, 2022), highlighting glaciers retreating (e.g., Marinelli) and advancing (e.g., Garibalid, Finlandia, Guilcher Oeste and Este). Subset of the center of the study region. Background image from Copernicus Sentinel data (2022-02-20).*

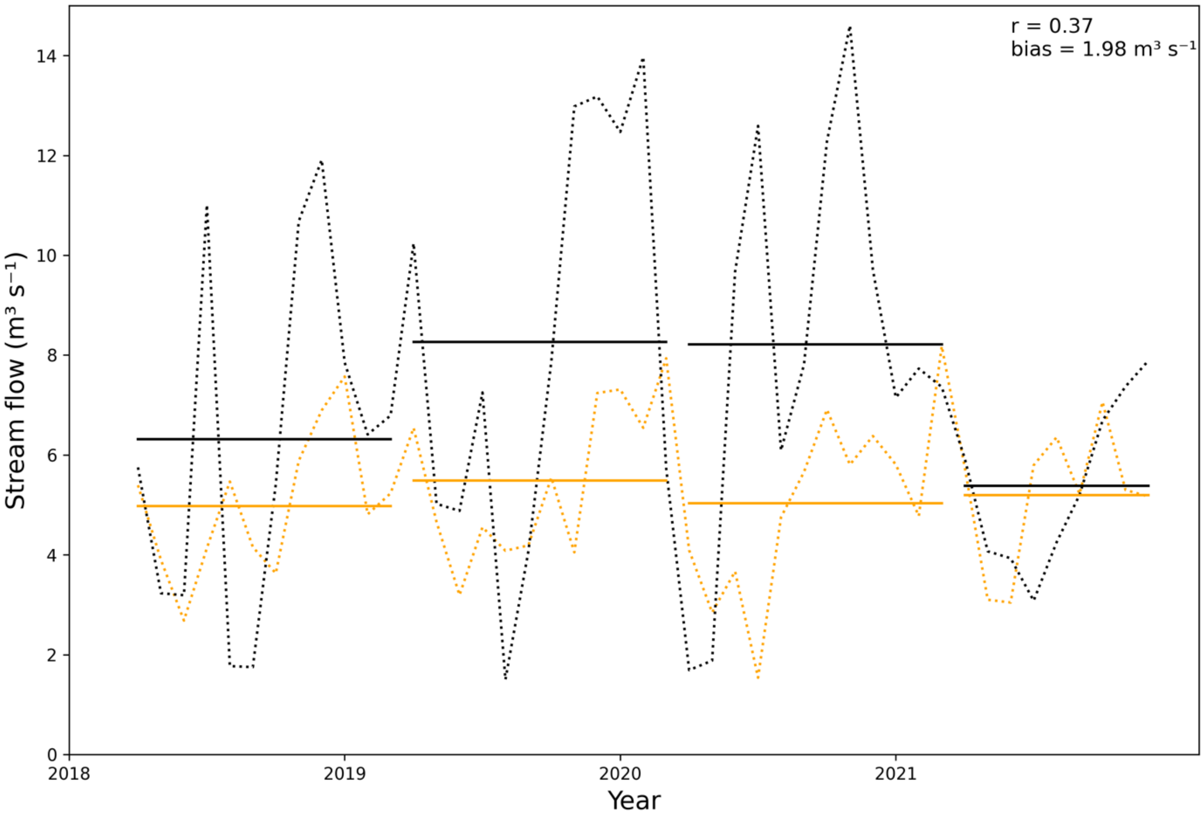

Figure S2: Comparison of modelled precipitation with runoff at Río Betbeder. *Water volume measured at the stream gauges (black) is compared to downscaled precipitation over the river catchment (orange) of Río Betbeder on monthly (dotted) and annual (solid) resolution. Pearson's correlation (r) and mean bias are given in the top right corner. Source data are provided as a Source Data file.*

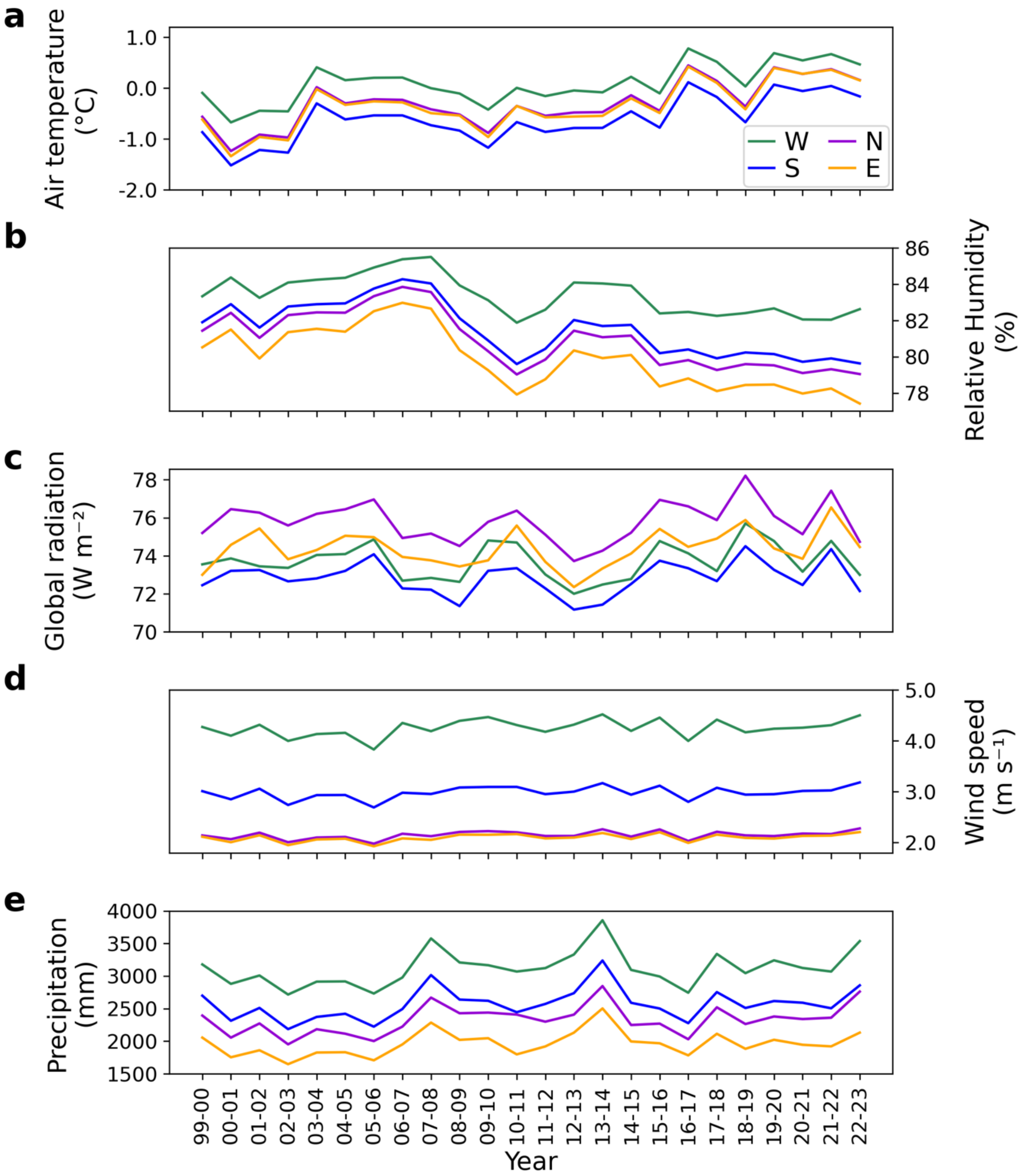

Figure S3: Regional climatic characteristics. *Mean annual* ***a*** *air temperature,* ***b*** *relative humidity,* ***c*** *global radiation,* ***d*** *wind speed and* ***e*** *precipitation for the four different subregions covering the period 1999-2022. Source data are provided as a Source Data file.*

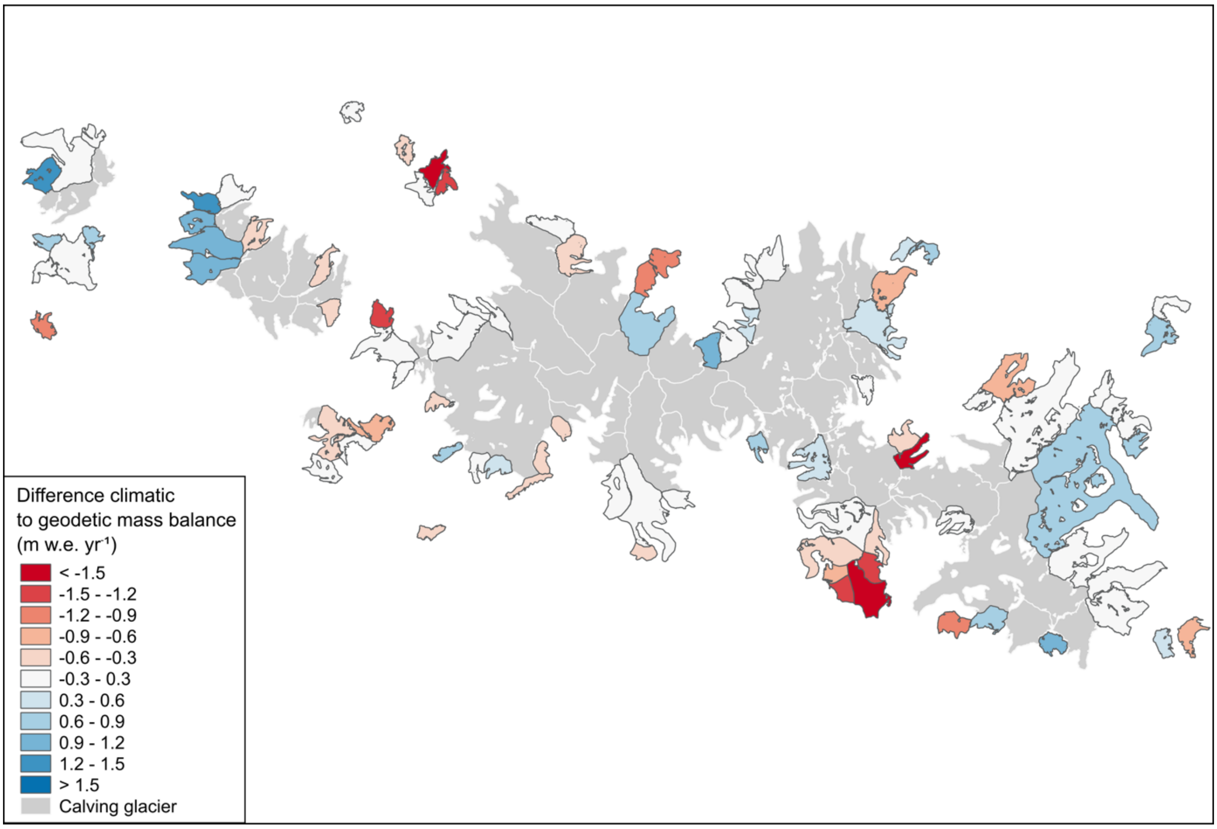

Figure S4: Climatic vs. geodetic mass balance. *Comparison of specific climatic and geodetic mass balance for land-terminating glaciers (> 3km^2) used for the model validation. The color scheme gives the difference between climatic and geodetic mass balance. Outlines display the glacier extent in 2000 (Barcaza et al., 2017).*

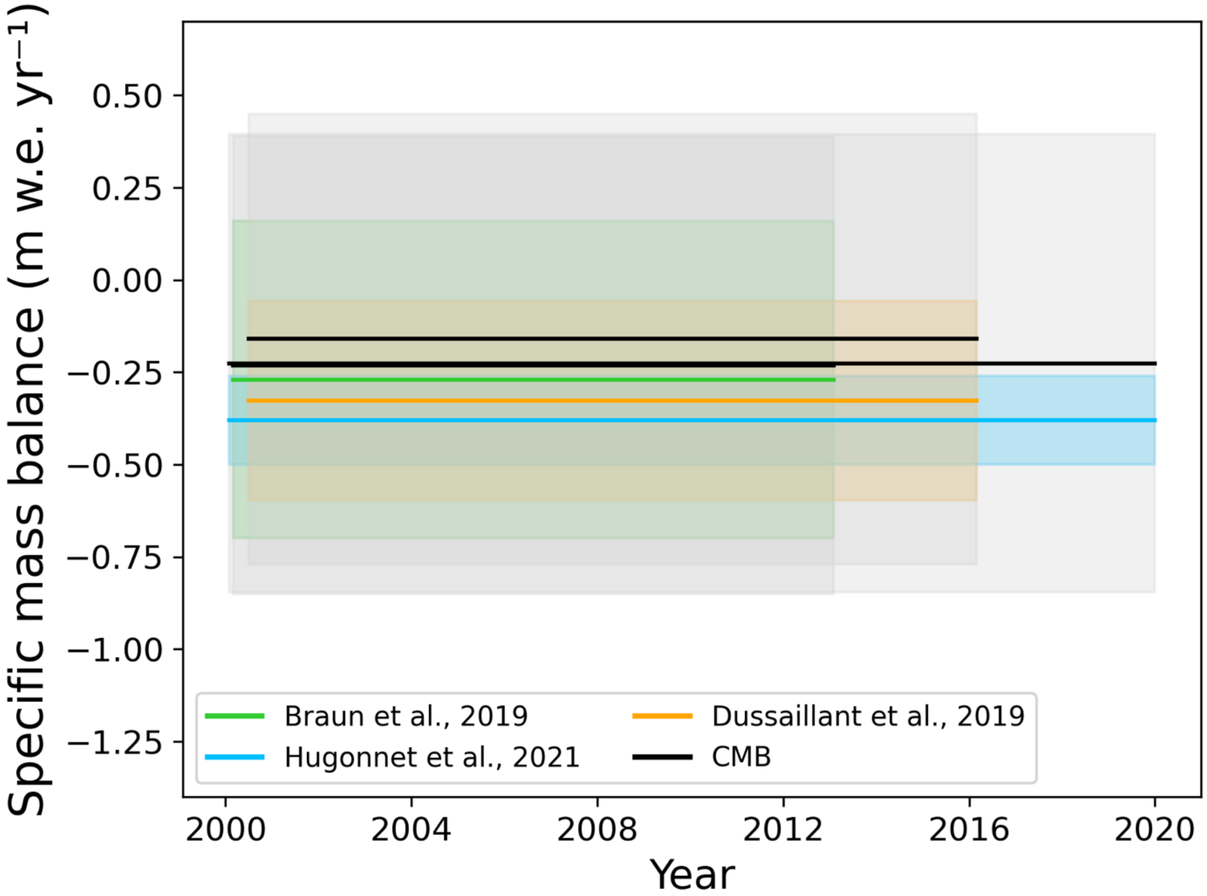

Figure S5: Mass change rates. *Comparison of mass change rates from climatic (this study) and multiple geodetic estimates for land-terminating glaciers (> 3 km^2) in the CDI. Source data are provided as a Source Data file.*

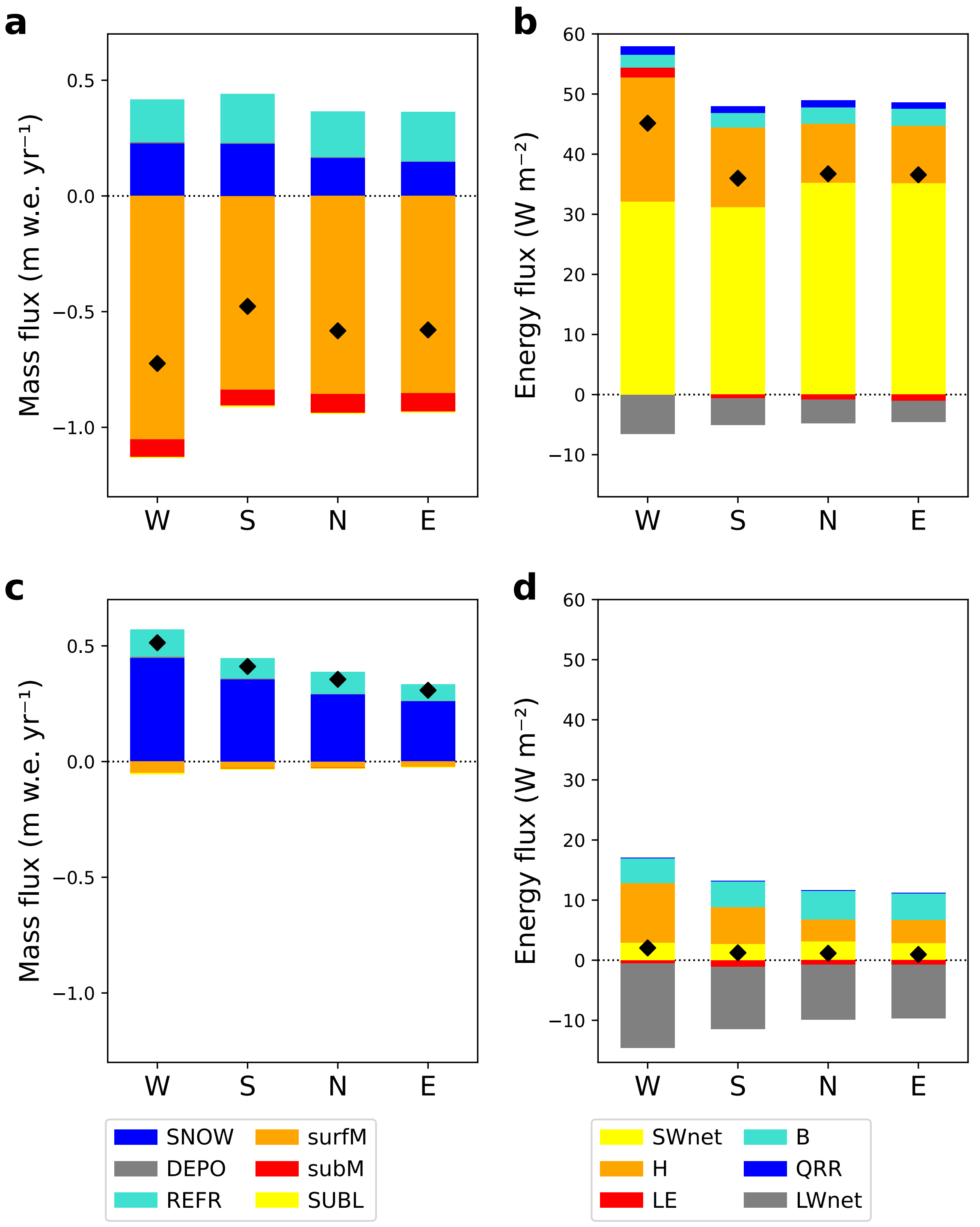

Figure S6: Seasonal energy and mass fluxes for the four subregions. *Average seasonal climatic mass (a, c) and energy (b, d) balance components for the four subdomains during austral summer (December, January, February) (a, b) and winter (June, Juli, August) (c, d): snowfall (SNOW), deposition (DEPO), refreezing (REFR), surface melt (surfM), subsurface melt (subM), sublimation (SUBL), net shortwave radiation (SWnet), sensible (H), latent (LE) and glacier heat flux (B), heat flux from rain (QRR) and net longwave radiation (LWnet).The black diamonds give the resulting climatic mass balance (a, c) and energy available for melting (b, d), respectively. Source data are provided as a Source Data file.*

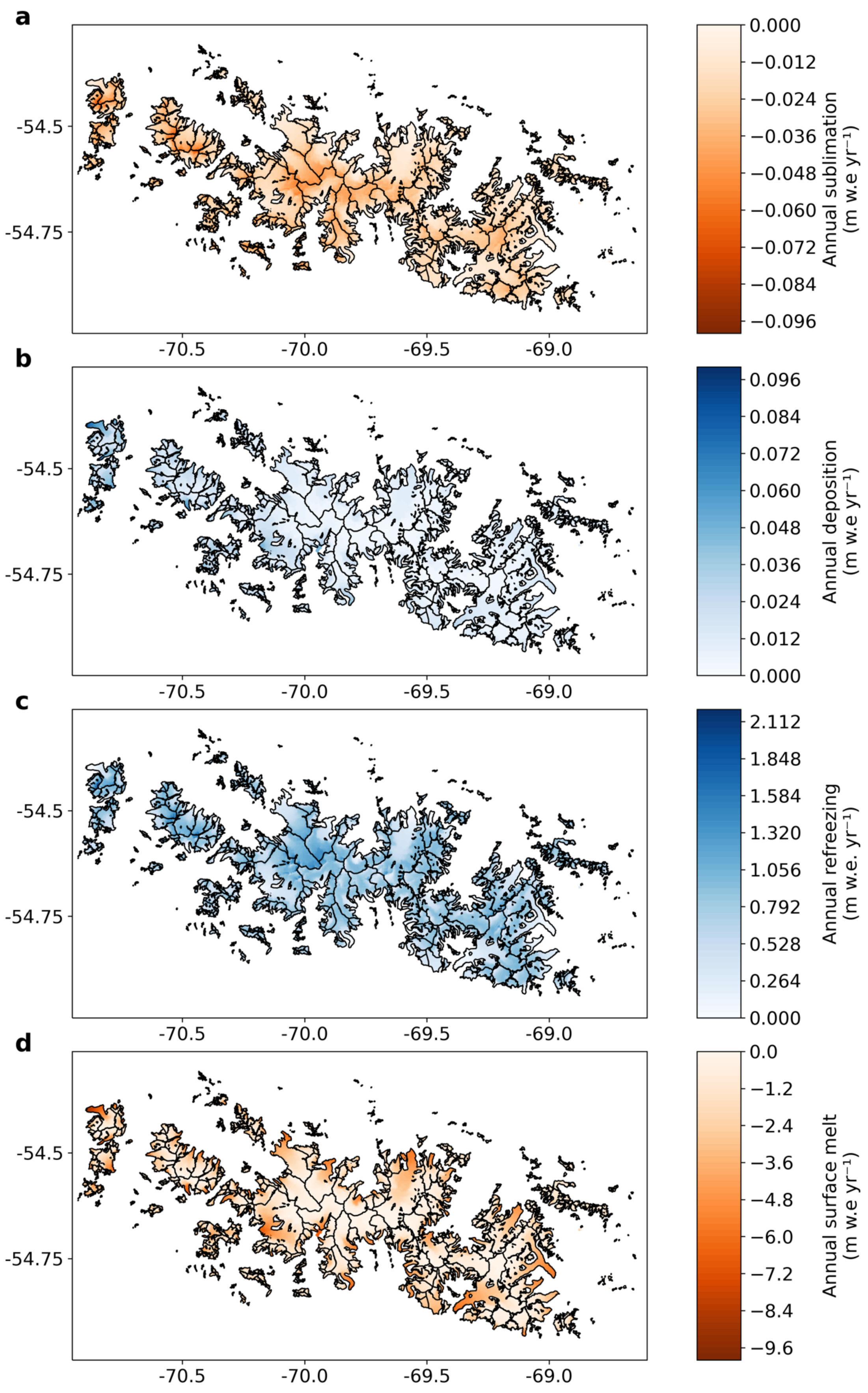

Figure S7: Climatic mass balance characteristics. *Panels show mean annual* ***a*** *sublimation,* ***b*** *deposition,* ***c*** *refreezing and* ***d*** *surface melt over the Cordillera Darwin Icefield (2000-2022) simulated with the COSIPY model. Black outlines display the glacier extent in 2000 (Barcaza et al., 2017).*

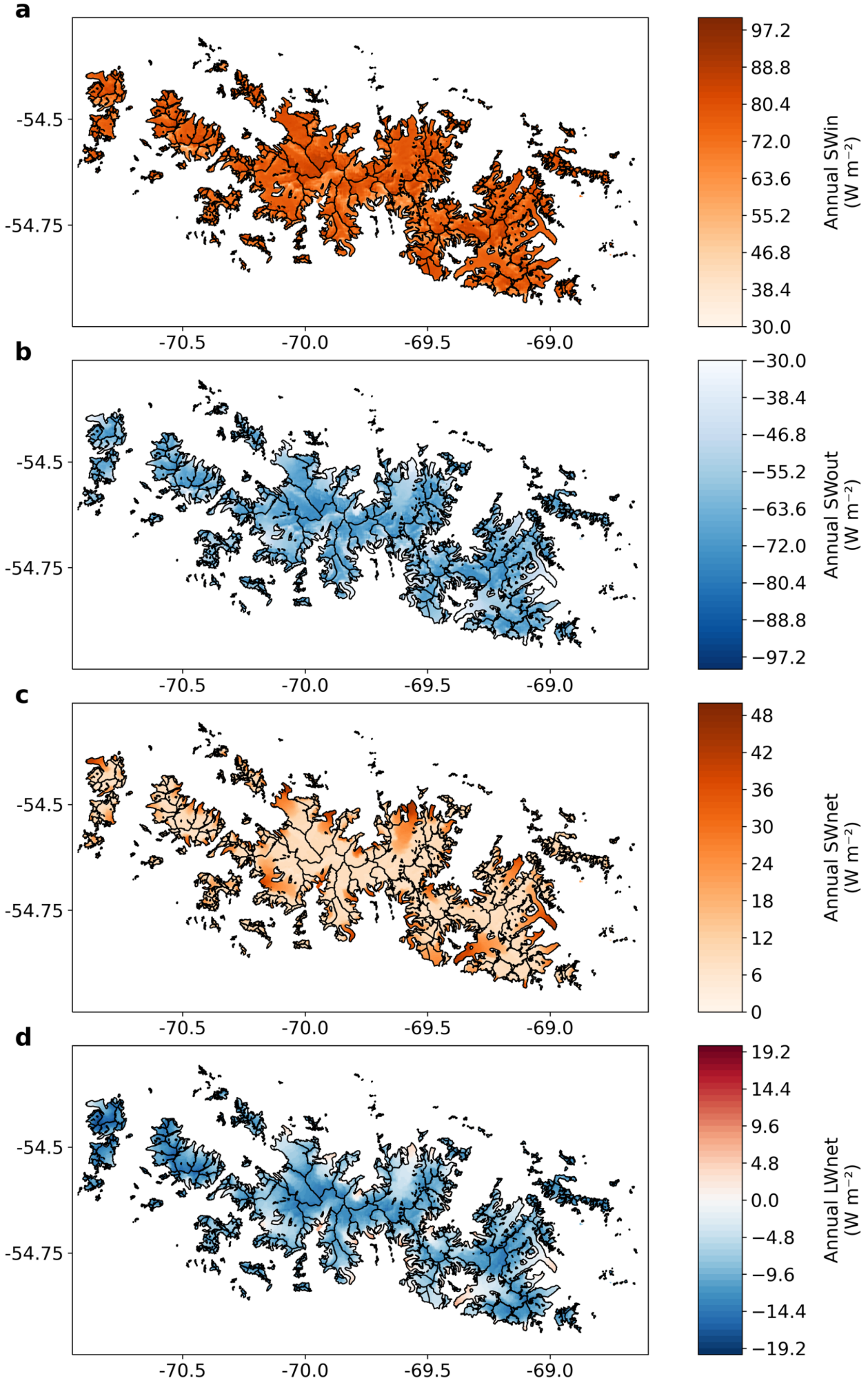

Figure S8: Climatic energy balance characteristics. *Panels show mean annual* ***a*** *incoming,* ***b*** *outgoing and* ***c*** *net shortwave radiation, and* ***d*** *net longwave radiation over the Cordillera Darwin Icefield (2000-2022) simulated with the radiation (a) and COSIPY (b-d) model. Black outlines display the glacier extent in 2000 (Barcaza et al., 2017).*

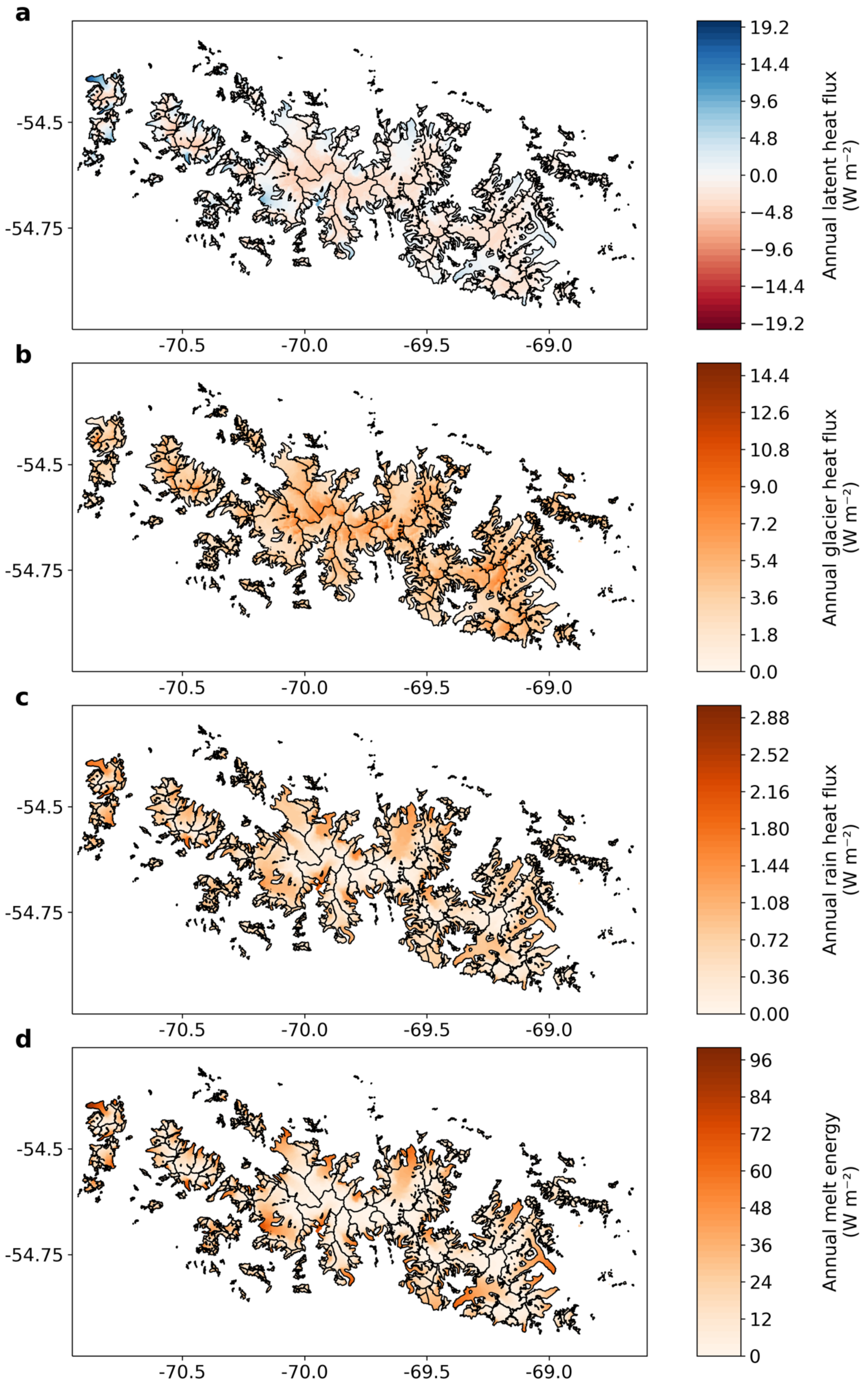

Figure S9: Climatic energy balance characteristics. *Panels show mean annual* ***a*** *latent,* ***b*** *glacier and* ***c*** *rain heat flux, as well as* ***d*** *melt energy over the Cordillera Darwin Icefield (2000-2022) simulated with the COSIPY model. Black outlines display the glacier extent in 2000 (Barcaza et al., 2017).*

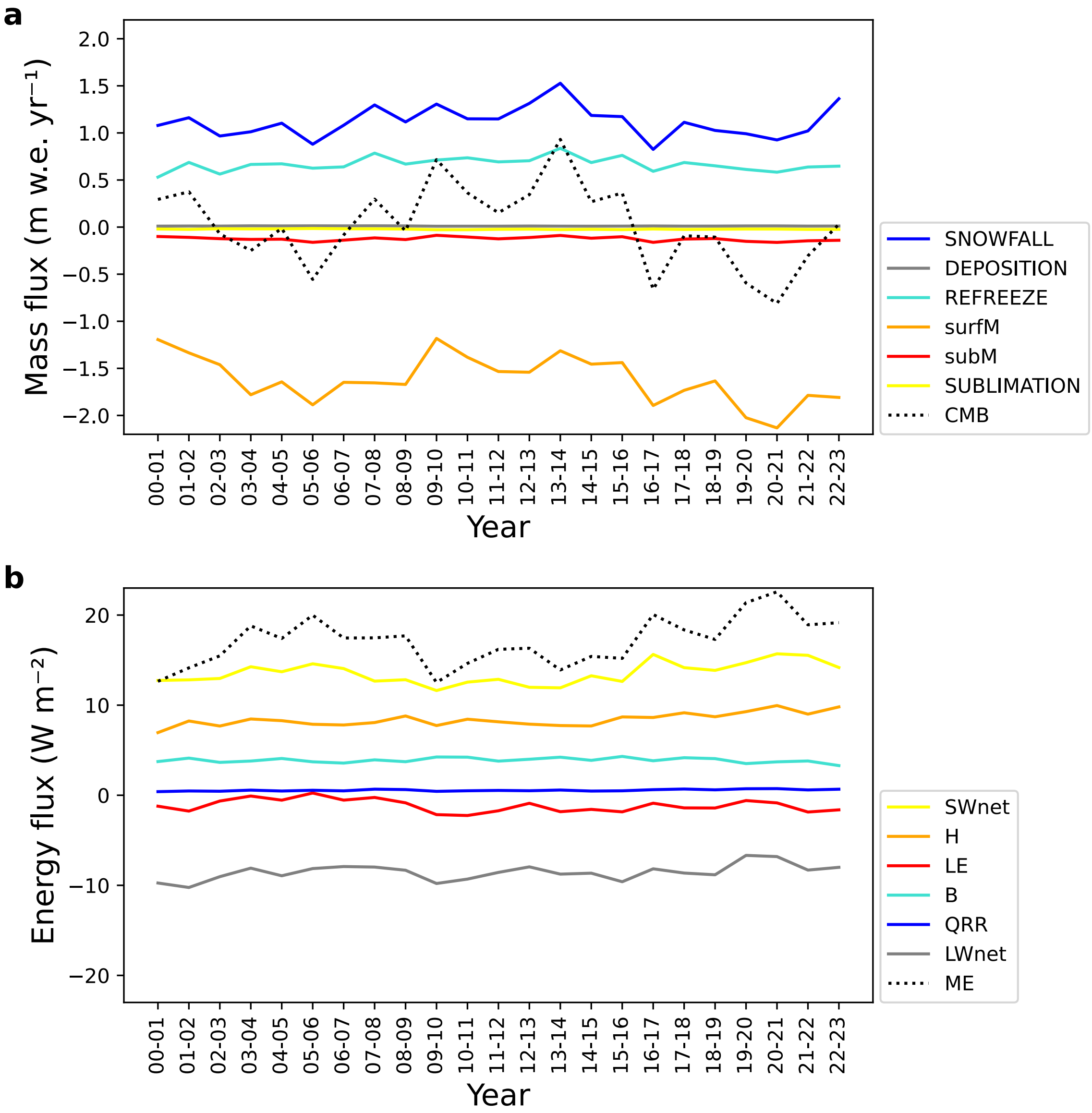

Figure S10: Annual climatic energy and mass fluxes. *Annual climatic mass (a) and energy (b) balance components: snowfall (SNOWFALL), deposition (DEPOSITION), refreezing (REFREEZE), surface melt (surfM), subsurface melt (subM), sublimation (SUBLIMATION), net shortwave radiation (SWnet), sensible (H), latent (LE) and glacier heat flux (B), heat flux from rain (QRR) and net longwave radiation (LWnet).The dotted lines give the resulting climatic mass balance (CMB) (a) and energy available for melting (ME) (b), respectively. Source data are provided as a Source Data file.*

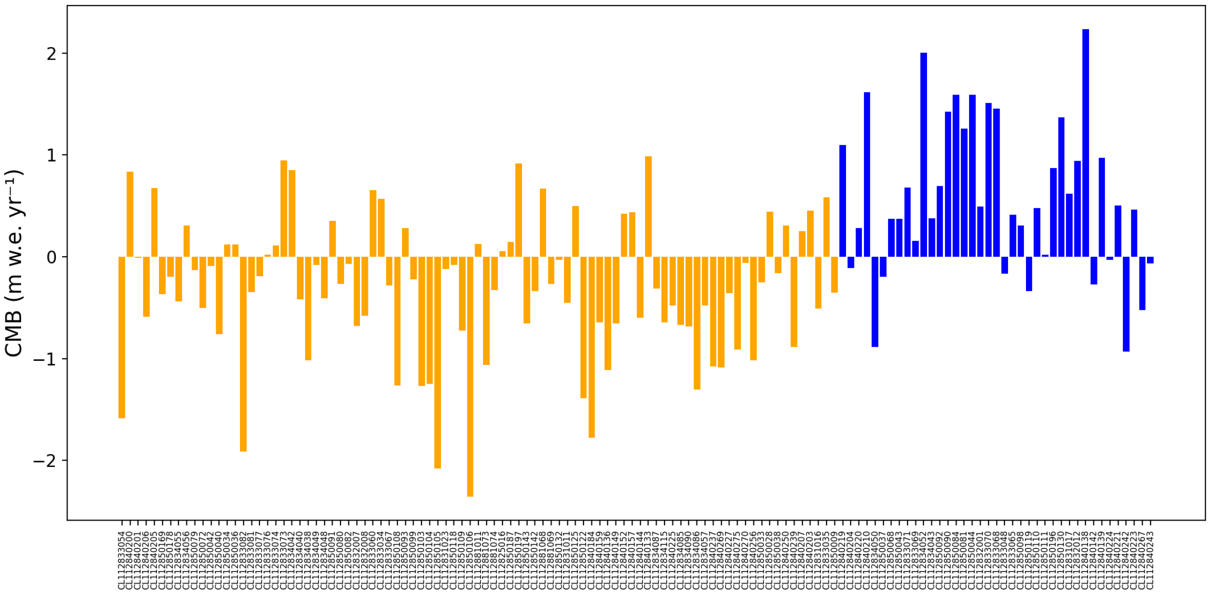

Figure S11: Average annual climatic mass balance per glacier (< 3 km²). *Color coding reveals the land-terminating (orange) and MALT (blue) glaciers. Source data are provided as a Source Data file.*

Supplementary Tables

Table S1: *Overview of the automatic weather stations (AWSs) used in this study. The variables (air temperature T, relative humidity RH, air pressure PRES, global radiation G, wind speed U, precipitation RRR and stream gauge runoff Q) are divided into their respective function in this study. Radiation and precipitation have been modeled while wind speed is directly taken from ERA5, thus all observations are used for validation. Stations that have been running over several periods give the respective variables during each period. At Schiaparelli there are two stations installed in close vicinity. Stations run by the Chilean Water Directorate (Dirección General de Aguas) give DGA as responsibility.*

Station name	Location (lat/lon)	Altitude (m a.s.l.)	Variables & function		Measurement period	Responsible
			Downscaling	Validation		
Schiaparelli	54.40°S 70.87°W	140	*T*, *RH*, *PRES*		08/2013 - 03/2020	J. Arigony-Neto & R. Jaña
		92		*G*, *U*, *RRR*	09/2015 - 01/2022	HU Berlin
Fiordo Agostini	54.48°S 70.47°W	20	*T*, *PRES*	*G*, *U*	04/2018 - 12/2022	J. Arigony-Neto
Bahía Pía	54.83°S 69.73°W	21	*T*, *RH*		02/2002 - 04/2003	E. Izaguirre
			PRES	*G*, *U*, *RRR*	02/2012 - 10/2015	
			T, *PRES*	*G*, *U*, *RRR*	04/2016 - 04/2018	
Marinelli	54.52°S 69.58°W	18		*T*	02/2014 - 05/2015	E. Izaguirre
				T	07/2017 - 11/2018	
Ainsworth	54.42°S 69.57°W	5		*T*, *PRES*, *G*, *U*, *RRR*	04/2011 - 07/2014	E. Izaguirre
Diablo	54.96°S 69.13°W	48	*T*		03/2011 - 03/2014	E. Izaguirre
Río Azorpado	54.50°S 68.82°W	32		*RRR*	03/2006 - 11/2022	DGA
Río Betbeder	54.56°S 68.82°W	60	*T*, *RH*	*RRR*, *Q*	11/2018 - 11/2022	DGA
Isla Hoste	54.95°S 68.70°W	10		*T*, *RH*, *PRES*, *G*, *U*, *RRR*	05/2019 - 08/2021	R. Giesecke
Bahía Yendegaia	54.88°S 68.68°W	10	*T*	*RRR*	06/2016 - 11/2022	DGA
Puerto Navarino	54.93°S 68.32°W	10	*T*, *RH*	*RRR*	12/2010 - 11/2022	DGA

Table S2: *Evaluation of daily downscaled climatic input data compared to the AWS observations. Statistics comprise mean model bias (MMB), root mean square error (RMSE) and Pearson's correlation (corr). Independent values are in black font while variables/stations used in the downscaling are in grey font.*

**Station name**	**T**			**RH**			**PRES**			**G**			**U**			**RRR**		
	MMB (°C)	RMSE (°C)	corr	MMB (%)	RMSE (%)	corr	MMB (hPa)	RMSE (hPa)	corr	MMB (W m^{-2})	RMSE (W m^{-2})	corr	MMB (m s^{-1})	RMSE (m s^{-1})	corr	MMB (mm)	RMSE (mm)	corr
Schiaparelli	0.36	0.85	0.95	4.04	7.25	0.67	5.57	7.28	0.99	-1.84	43.49	0.73	-1.15	1.70	0.75	57.22	66.95	0.86
Fiordo Agostini	-0.11	1.04	0.94				-0.87	4.55	1.00	18.32	42.36	0.87	-2.02	2.24	0.70			
Bahía Pía	0.01	1.38	0.92	-0.11	8.07	0.64	-4.71	6.74	1.00				5.62	6.70	0.74	34.51	43.77	0.74
Marinelli	1.16	2.09	0.84															
Ainsworth	0.03	1.30	0.92				-4.47	6.45	0.99				6.71	8.29	0.68	25.55	41.81	0.66
Diablo	0.53	1.35	0.93															
Río Azorpado																22.47	53.85	0.33
Río Betbeder	0.46	1.50	0.95	2.82	7.23	0.80										71.32	75.16	0.65
Isla Hoste	-0.10	1.17	0.97	-0.25	7.08	0.64	-2.78	5.67	0.99				2.59	3.43	0.77	26.04	33.18	0.72
Bahía Yendegaia	0.37	1.49	0.94															
Puerto Navarino	0.16	1.31	0.95	-3.50	7.88	0.73										0.56	16.04	0.57

Table S3: *Confidence levels as defined in the IPCC guidance for communication of confidence (IPCC, 2022).*

**Likelihood Language**	**Statistical confidence level**
Virtually certain	>99%
Extremely likely	>95%
Very likely	>90%
Likely	>66%

Table S4: *Total (A_{tot}), climatic (A_{clim}) and frontal ablation (A_{f_budget} for mass budgeting and A_{f_FG} the flux gate) for the marine- and lake-terminating (MALT) glaciers of the Cordillera Darwin Icefield (2000-2013), all in Gt yr^{-1}. The fraction of frontal to total ablation (FF) for the mass budgeting approach is given in the last column.*

ID	Name	A_{tot}	A_{clim}	A_{f_budget}		A_{f_FG}		FF
CL112834052	Rugidor	0.15	0.03	0.11	±0.04	0.13	±0.04	0.78
CL112850130	Italia	0.04	0.01	0.02	±0.01	0.04	±0.01	0.71
CL112832001	Darwin	0.20	0.06	0.14	±0.04	0.14	±0.05	0.70
CL112833069	Marinelli	0.57	0.18	0.40	±0.10	0.23	±0.09	0.69
CL112833070		0.06	0.02	0.04	±0.02	0.02	±0.01	0.68
CL112840210		0.02	0.01	0.01	±0.01	0.00	±0.00	0.67
CL112840138		0.02	0.01	0.01	±0.00	0.00	±0.00	0.65
CL112850090	Guilcher Este	0.03	0.01	0.02	±0.01	0.01	±0.00	0.62
CL112850084	Guilcher Oeste	0.07	0.03	0.04	±0.03	0.06	±0.02	0.60
CL112833068		0.03	0.01	0.02	±0.01	0.03	±0.01	0.59
CL112850037	Grande	0.54	0.24	0.30	±0.10	0.17	±0.06	0.55
CL112850081		0.11	0.05	0.06	±0.04	0.07	±0.02	0.55
CL112832012	Cuevas	0.08	0.04	0.04	±0.04	0.03	±0.01	0.52
CL112840139	Lovisato	0.05	0.03	0.02	±0.01	0.02	±0.01	0.48
CL112850196	Frances	0.03	0.02	0.01	±0.01	0.02	±0.01	0.41
CL112831017		0.03	0.02	0.01	±0.01	0.02	±0.01	0.39
CL112840219		0.06	0.04	0.02	±0.02	0.06	±0.01	0.38
CL112840221		0.04	0.02	0.01	±0.01	0.05	±0.01	0.38
CL112850044	Garibaldi	0.10	0.06	0.04	±0.05	0.09	±0.04	0.37
CL112850068		0.02	0.01	0.01	±0.01	0.01	±0.00	0.34
CL112840220		0.04	0.03	0.01	±0.01	0.00	±0.01	0.33
CL112850110		0.02	0.01	0.00	±0.01	0.00	±0.00	0.26
CL112850098		0.02	0.02	0.01	±0.01	0.01	±0.01	0.24
CL112850119	Alemania	0.28	0.22	0.06	±0.09	0.05	±0.03	0.23
CL112834043	Relander	0.10	0.08	0.02	±0.04	0.08	±0.02	0.22
CL112840204		0.02	0.02	0.01	±0.01	0.01	±0.00	0.21
CL112850092		0.03	0.03	0.00	±0.02	0.02	±0.00	0.15
CL112833065		0.02	0.02	0.00	±0.01	0.01	±0.01	0.13
CL112833071	Finlandia	0.04	0.03	0.00	±0.02	0.06	±0.02	0.11
CL112840142		0.02	0.02	0.00	±0.01	0.00	±0.00	0.07
CL112833048		0.04	0.04	0.00	±0.02	0.01	±0.00	0.04
CL112840267		0.01	0.01	0.00	±0.00	0.00	±0.00	0.03
CL112840223		0.01	0.01	0.00	±0.01	0.00	±0.00	0.02
CL112850111		0.01	0.01	0.00	±0.01	0.01	±0.00	0.00
CL112840243		0.00	0.01	0.00	±0.00	0.00	±0.00	0.00
CL112840224		0.02	0.02	0.00	±0.01	0.02	±0.01	0.00
CL112834050		0.10	0.11	0.00	±0.04	0.01	±0.00	0.00
CL112850043		0.01	0.01	0.00	±0.00	0.00	±0.00	0.00
CL112840242		0.01	0.01	0.00	±0.00	0.01	±0.00	0.00
MALT total		3.02	1.57	1.44	±0.94	1.49	±0.53	0.48

Table S5: *Overview of the applied model set-up.*

	**Parameter**	**Value/Method**	**Unit**	**Source**
Model set-up	Temporal resolution	3	hour	-
	Spatial resolution	200	m	-
Climatic forcing	Temperature lapse rate	-0.60	K 100 m^{-1}	Schaefer et al. (2015); Weidemann et al. (2020); Buttstädt et al. (2009); Koppes et al. (2009); Bown et al. (2019)
	Conversion/fallout time of hydrometeors	1200	s	Barstad and Smith (2005); Jarosch et al. (2012); Sauter (2020); Schuler et al. (2008); Smith (2003)
	Relative humidity threshold	90	%	Temme et al. (2023)
COSIPY	Stability correction	Monin-Obukhov	-	Temme et al. (2023)
	Center snow transfer function	1.0	°C	Temme et al. (2023)
	Spread snow transfer function	1.0	-	Temme et al. (2023)
	Albedo fresh snow	0.9	-	Oerlemans and Knap, 1998
	Albedo firn	0.5	-	Temme et al. (2023)
	Albedo ice	0.3	-	Temme et al. (2023)
	Time constant snow albedo ageing	22	day	Temme et al. (2023)
	Depth constant snow albedo ageing	3	cm	Temme et al. (2023)
	Roughness length fresh snow	0.24	mm	Mölg et al., 2012
	Roughness length firn	4.0	mm	Mölg et al., 2012
	Roughness length ice	0.3	mm	Temme et al. (2023)

Table S6: *Evaluation of modelled glacier ablation compared to observations. Statistics comprise mean model bias (MMB), root mean square error (RMSE) and Pearson's correlation (corr).*

	**Schiaparelli**	**Martial Este**
Measurement period	2013 - 2020	2000 - 2020
MMB (m w.e. yr^{-1})	0.25	-0.68
RMSE (m w.e. yr^{-1})	1.86	0.81
corr	0.42	0.74
